# MicroRNAs in atrial fibrillation – have we discovered the Holy Grail or opened a Pandora’s box?

**DOI:** 10.3389/fphar.2025.1535621

**Published:** 2025-02-12

**Authors:** Alkora Ioana Balan, Alina Scridon

**Affiliations:** Physiology Department and Center for Advanced Medical and Pharmaceutical Research, Pharmacy, Science and Technology “George Emil Palade” of Târgu Mureș, University of Medicine, Târgu Mures, Romania

**Keywords:** antiarrhythmic therapy, atrial fibrillation, electrical remodeling, microRNA, structural remodeling

## Abstract

Atrial fibrillation (AF) causes a heavy socio-economic burden on healthcare systems around the globe. Identification of new preventive, diagnostic, and treatment methods is imperative. In recent years, special attention has been paid to microRNAs (miRNAs) as potential regulators of AF pathogenesis. Through post-transcriptional regulation of genes, miRNAs have been shown to play crucial roles in AF-related structural and electrical atrial remodeling. Altered expression of different miRNAs has been related to proarrhythmic changes in the duration of action potentials and atrial fibrosis. In clinical studies, miRNA changes have been associated with AF, whereas in experimental studies miRNA manipulation has emerged as a potential therapeutic approach. It would appear that, with the advent of miRNAs, we may have found the Holy Grail, and that efficient and personalized AF therapy may be one step away. Yet, the clinical relevance of miRNA evaluation and manipulation remains questionable. Studies have identified numerous miRNAs associated with AF, but none of them have shown sufficient specificity for AF. MicroRNAs are not gene-specific but regulate the expression of a myriad of genes. Cardiac and non-cardiac off-target effects may thus occur following miRNA manipulation. A Pandora’s box might thus have opened with the advent of these sophisticated molecules. In this paper, we provide a critical analysis of the clinical and experimental, epidemiological and mechanistic data linking miRNAs to AF, we discuss the most promising miRNA therapeutic approaches, we emphasize a number of questions that remain to be answered, and we identify hotspots for future research.

## 1 Introduction

Atrial fibrillation (AF) is the most common sustained cardiac arrhythmia, affecting ≈33 million patients worldwide, and is associated with considerable morbidity, mortality and, quality of life impairment (Kornej et al., 2020). Numerous risk factors have been associated with the occurrence and progression of AF. Among them, heart failure, diabetes, hypertension, obesity, and structural and ischemic heart disease are strong predictors of AF (Kornej et al., 2020). Several biomarkers, such as C-reactive protein, cardiac troponins, B-type natriuretic peptide, and components of the renin-angiotensin-aldosterone system (RAAS) have also been associated with AF (Kornej et al., 2020). However, none of these risk factors or biomarkers have proven enough reliability in predicting future AF and none of them are specific for AF. Multimarker strategies and risk scores have also been proposed as potential approaches for AF prediction. However, none of them have managed to prove their efficacy in clinical trials (Olivia et al., 2019). Moreover, AF can also occur in individuals devoid of overt risk factors for AF (Olivia et al., 2019; Fan et al., 2019).

MicroRNAs (miRNAs) are short non-coding RNAs that play critical roles in post-transcriptional regulation of gene expression (Yang et al., 2007; Soeki et al., 2016; Li et al., 2014). Several miRNAs have been associated with cardiac pathologies via regulation of genes involved in inflammation, myocardial remodeling, apoptosis, and cardiac ions dynamics (Yang et al., 2007; Soeki et al., 2016; Li et al., 2014). Recent research has demonstrated an association between different cardiac and circulating miRNAs and atrial structural, electrical, and autonomic remodeling, suggesting that they could serve as biomarkers for AF diagnostic and/or prediction, as well as potential therapeutic targets for AF (Yang et al., 2007; Soeki et al., 2016; Li et al., 2014). If confirmed, these hypotheses could radically change the AF landscape. However, none of those miRNAs are specific for AF (Yang et al., 2007; Soeki et al., 2016; Li et al., 2014) and to date, no study has assessed the therapeutic potential of miRNA manipulation for AF in clinical settings.

This article aims to provide a critical analysis of the most relevant clinical and experimental, epidemiological and mechanistic data linking miRNAs to AF and to discuss the potential role of miRNAs as biomarkers for diagnosis, prognosis and monitoring of AF, as well as therapeutic targets in AF. We also emphasize several questions that remain to be answered and we identify hotspots for future research. To highlight the role of miRNAs in AF, the miRNAs that provide a scientifically comprehensive overview and have practical importance in advancing the management and understanding of AF were selected. miRNAs supported by strong evidence were prioritized, highlighting those extensively documented in both experimental and clinical studies as having significant roles in AF pathophysiology. In addition, the clinical applicability of these miRNAs was considered, focusing on those miRNAs that have demonstrated potential as diagnostic markers, prognostic tools, or therapeutic targets in clinical settings.

## 2 MicroRNAs in atrial fibrillation

**FIGURE 1:**
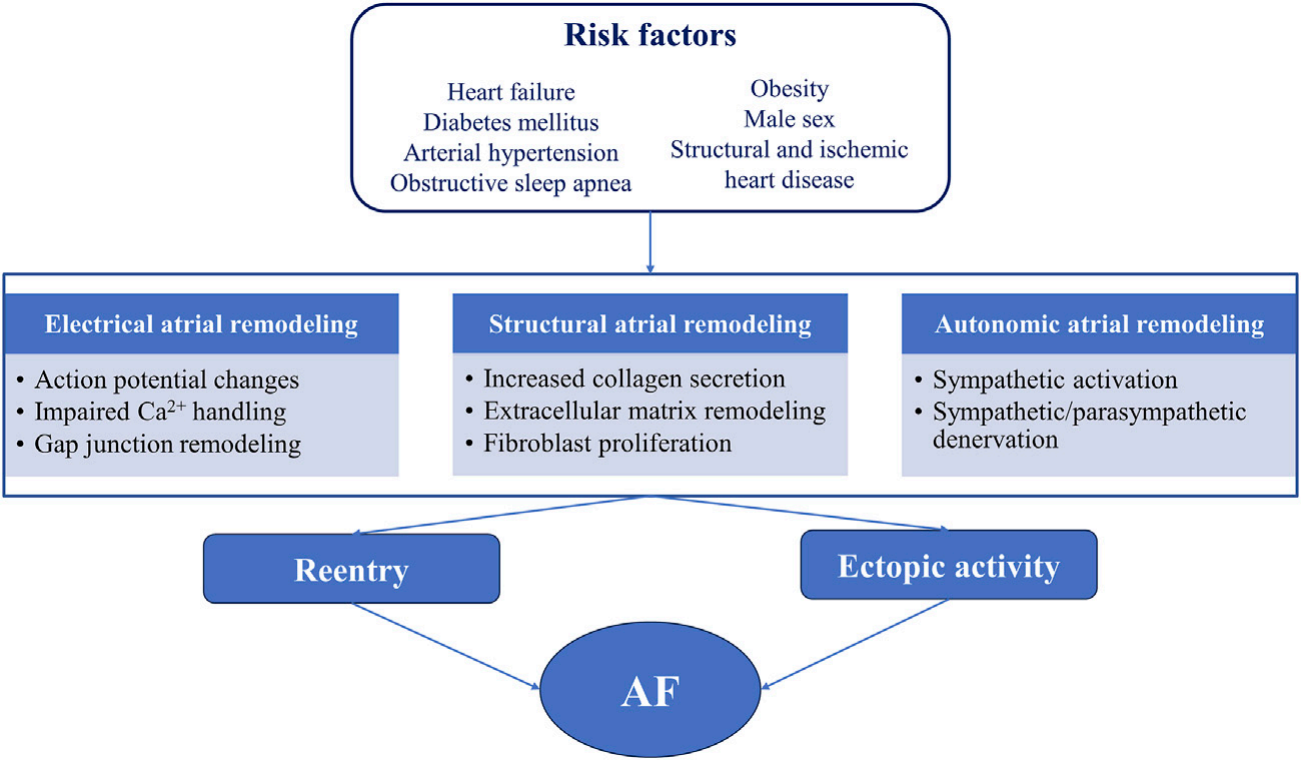
Relationship between common risk factors, atrial proarrhythmic remodeling, and atrial fibrillation AF, atrial fibrillation.

**FIGURE 2:**
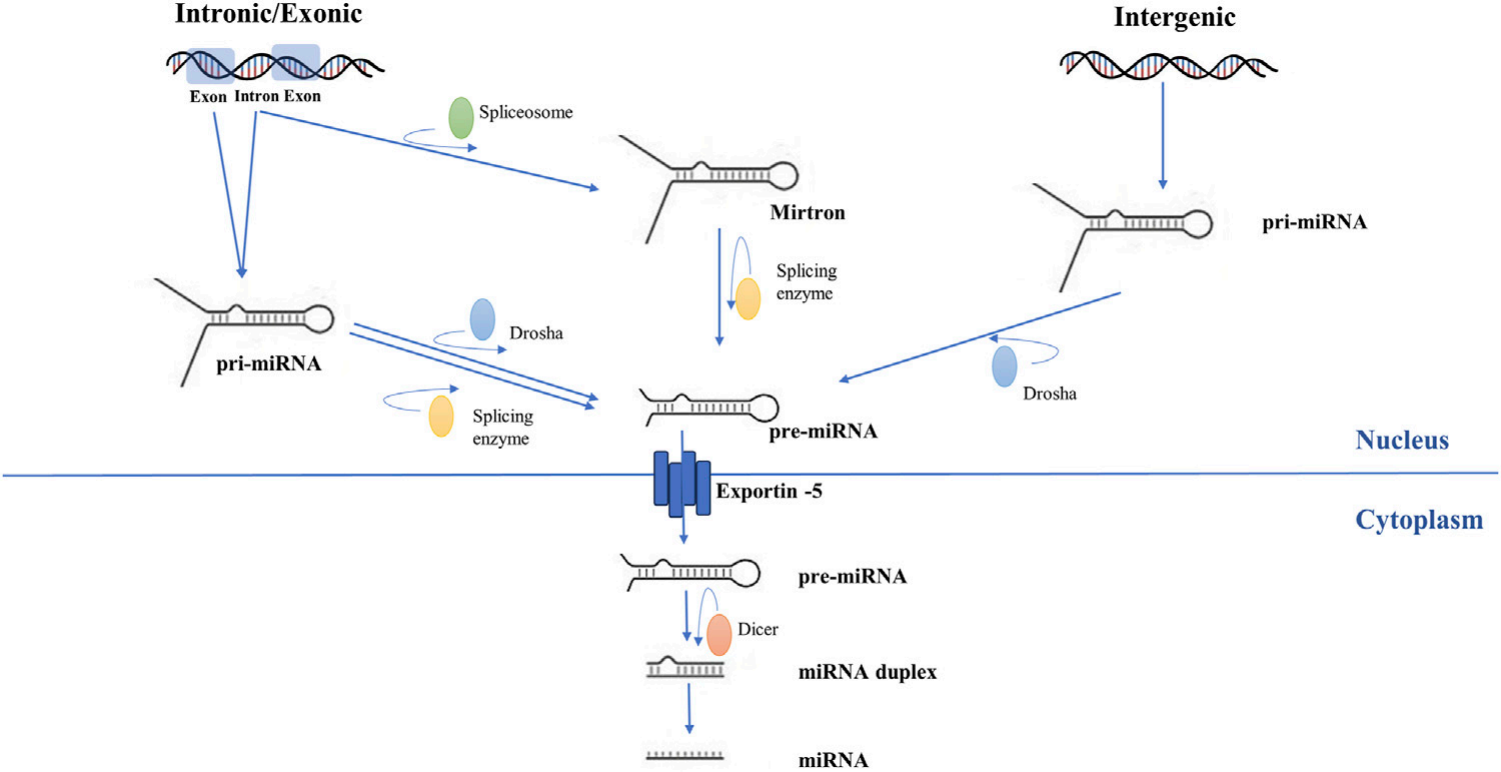
Schematic representation of microRNA biogenesis. Genomic DNA is transcribed into pri-miRNA by RNA polymerase II. Subsequently, endonucleotide cleavage of the pri-miRNA generates a miRNA precursor (pre-miRNA). For intergenic miRNAs, this process is carried out exclusively by ribonuclease 3. For intronic and exonic miRNAs, pre-miRNA formation involves further modulation by splicing enzymes. Pre-miRNA is exported to the cytoplasm. Dicer enzyme processes pre-miRNA and transforms it into duplex miRNA with two arms, of which one will become a mature miRNA and the other will be eliminated.

The pathophysiological basis of AF involves complex mechanisms (Figure 1) (Șerban et al., 2019; Scridon et al., 2018). The biogenesis and mechanism of action of miRNAs (Figure 2) and the potential role of miRNAs as biomarkers and therapeutic targets in cardiovascular diseases have been intensively studied over the past years (Yang et al., 2007; Soeki et al., 2016; Li et al., 2014).

**FIGURE 3:**
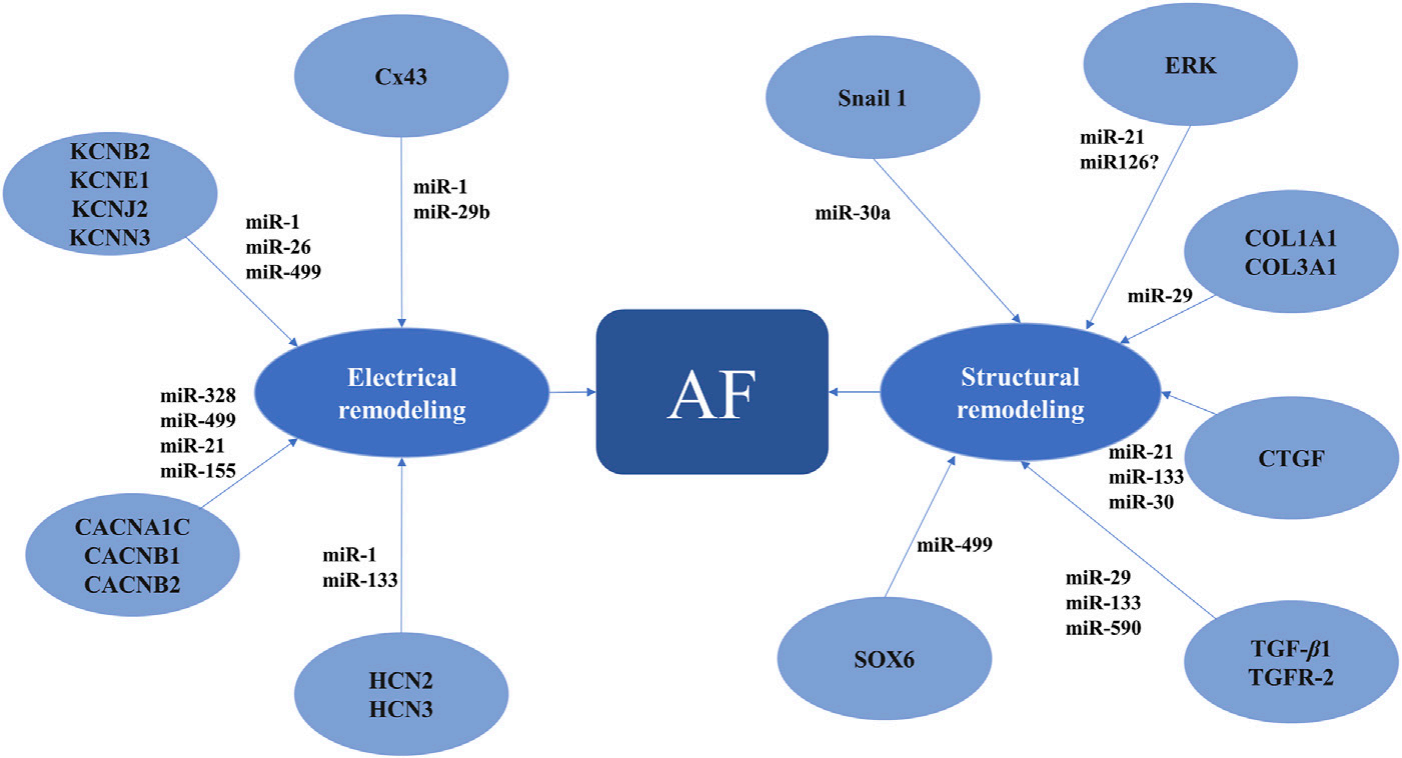
MicroRNAs involved in proarrhythmic atrial electrical and structural remodeling AF, atrial fibrillation; CACNA1C, calcium voltage-gated channel subunit alpha1 C; CACNB1, calcium voltage-gated channel auxiliary subunit beta1; CACNB2, calcium voltage-gated channel auxiliary subunit beta2; COL1A1, collagen type I alpha1 chain; COL3A1, collagen type III alpha1 chain; CTGF, connective tissue growth factor; Cx43, connexin 43; ERK, extracellular regulated mitogen-activated protein kinase; HCN2, 3, hyperpolarization activated cyclic nucleotide gated potassium and sodium channel 2, 3; KCNB2, potassium voltage-gated channel subfamily B member 2; KCNE1, potassium voltage-gated channel subfamily E regulatory subunit 1; KCNJ2, potassium inwardly rectifying channel subfamily J member 2; KCNN3, potassium calcium-activated channel subfamily N member 3; SOX6, SRY-box transcription factor 6; TGF-β1, transforming growth factor beta1; TGFR-2, transforming growth factor beta receptor 2.

In AF, miRNAs have been associated with changes in the expression of genes involved in cardiac function and atrial tissue remodeling (Figure 3). Experimental and clinical studies identified several miRNAs that are upregulated or downregulated in AF (Yang et al., 2007; Soeki et al., 2016; Li et al., 2014). Changes in miRNA levels were observed not only in the atrial tissue but also in the peripheral blood, offering the possibility of using them as readily available biomarkers for the prediction and/or diagnosis of AF (Yang et al., 2007; Soeki et al., 2016; Li et al., 2014). In addition, the causal link between AF and certain miRNAs suggests that manipulation of miRNA expression could represent a novel therapeutic strategy in AF (Yang et al., 2007).

### 2.1 MicroRNA-mediated atrial electrical remodeling

#### 2.1.1 miR-1

The first studies that demonstrated the role of miR-1 in cardiac electrophysiology were performed in the setting of cardiac ischemia, where miR-1 overexpression was shown to contribute to cardiac arrhythmogenesis (Yang et al., 2007). In experimental studies, ventricular miR-1 overexpression increased spontaneous Ca2+ release from the sarcoplasmic reticulum, decreased inward Ca2+ current amplitude, and increased RyR2 activity, leading to Ca2+ handling abnormalities, intracellular Ca2+ overload, and arrhythmias (Terentyev et al., 2009). These changes were accompanied by post-transcriptional repression of KCNJ2 (Yang et al., 2007). Decreased conductance induced by miR-1 overexpression was attributed to post-transcriptional repression of GJA1, encoding for connexin 43 (Cx43), a key component of gap junctions, which are essential for intercellular conduction in the ventricles (Yang et al., 2007). Meanwhile, miR-1 knockdown reduced ischemia-induced arrhythmias, suggesting the potential of miR-1 manipulation as antiarrhythmic strategy (Yang et al., 2007). Considering the importance of Ca2+, GJA1/Cx43, and KCNJ2/Kir2.1/IK1 in atrial electrophysiology, subsequent studies focused on the possible involvement of miR-1 in AF.

In patients with AF, plasma levels of miR-1 were higher in the left atrium than in the pulmonary veins, suggesting that local left atrial production of miR-1 might be involved in proarrhythmic atrial remodeling (Soeki et al., 2016). However, the exact changes that occur in miR-1 in AF remain controversial. Left atrial miR-1 levels were significantly higher in patients who developed post-coronary bypass AF than in those who did not, but this was not reflected in the peripheral blood (Tsoporis et al., 2018). On the contrary, in the study by Girmatsion et al., atrial miR-1 expression was reduced by ≈ 86% in patients with persistent AF compared to sinus rhythm controls (Girmatsion et al., 2009). A progressive decrease in miR-1 expression with advancing age (Li et al., 2015) may explain at least some of these discrepancies.

The exact mechanisms by which miR-1 changes lead to atrial electrical remodeling and AF are also incompletely elucidated. In patients with persistent AF, reduced atrial miR-1 levels were associated with an increase in atrial KCNJ2, Kir2.1, and, IK1 density (Girmatsion et al., 2009). Reduced miR-1 levels were also accompanied by increased HCN2 and HCN4 channels expression in elderly patients with AF (Li et al., 2015). Although miR-1-dependent gene regulation seems to be involved in Ca2+ homeostasis regulation in the ventricles (Terentyev et al., 2009), evidence of this regulatory mechanism extending to atrial fibrillation remains unestablished. The potential of miR-1 manipulation as a therapeutic target in AF was tested in a model of programmed atrial electrical stimulation, where right atrial tachypacing led to miR-1 upregulation and KCNE1 and KCNB2 downregulation (Jia et al., 2013). Contrary to expectations, these were accompanied by an increase in IKs activity and a decrease in atrial efective refractory period (AERP) duration (Jia et al., 2013). Additional miR-1 upregulation led to further amplification of these molecular and electrical changes. In contrast, administration of locked nucleic acids-based antimiR-1 prolonged the AERP and reduced AF susceptibility and duration in rabbits, supporting the potential role of miR-1 as a therapeutic target in AF (Jia et al., 2013).

miR-1 expression is not limited, however, to tissues directly involved in AF, and altered miR-1 expression has also been reported in many other cardiac and non-cardiac diseases (Yang et al., 2007; Ma et al., 2020; De Gonzalo-Calvo et al., 2017; Sugiyama et al., 2020). Thus, miR-1 manipulation to prevent AF could have off-target effects, whereas the potential use of miR-1 as a biomarker for AF, as proposed in several studies (Yang et al., 2007; Soeki et al., 2016), is considerable affected by its low specificity (Li et al., 2014). In addition, various physiological and pathological conditions make miR-1 expression unstable over time.

#### 2.1.2 miR-328

In a study evaluating peripheral, pulmonary vein, and left atrial appendage plasma levels of miR-328 in patients with and without AF, no significant differences were observed in miR-328 plasma levels between patients with AF and those in sinus rhythm. However, intracardiac levels of miR-328 were increased in AF patients compared to their peripheral and pulmonary vein plasma levels, suggesting that altered left atrial miR-328 expression is involved in atrial remodeling (Soeki et al., 2016). We have recently shown in a rat model of AF a strong association between increased miR-328 levels and AF, suggesting that miR-328 could emerge as a promising biomarker for AF diagnosis (Scridon et al., 2023). Moreover, in that model, the increase in miR-328 preceded the appearance of the arrhythmia, suggesting that dynamic miR-328 monitoring may predict future AF. Similar associations between increased miR-328 levels and AF were also reported in clinical studies (Huang et al., 2022).

Using a computational prediction algorithm, Lu et al. identified CACNA1C and CACNB1, encoding the α1C- and β1-subunits of ICa-L, respectively, as potential targets for miR-328 (Lu et al., 2010). This was further confirmed by Western blot and luciferase activity assay (Lu et al., 2010). The authors subsequently demonstrated that miR-328 overexpression increased AF susceptibility in dogs and mice, probably due to decreased ICa-L and AERP shortening (Lu et al., 2010). Contrarily, antagomiR-328 reversed those changes and decreased AF susceptibility (Lu et al., 2010).

However, miR-328 expression is not changed only in AF. Down- or upregulation of miR-328 has been reported in a series of primary tumors and metastases (Wang et al., 2023). Altering miR-328 levels may thus disrupt other physiological functions and lead to unwanted effects, potentially increasing the risk of neoplasia (Wang et al., 2023).

#### 2.1.3 miR-499

miR-499 was overexpressed in the atrial tissue of patients with AF, compared to patients in sinus rhythm (Ling et al., 2017). In those patients, miR-499 overexpression was linked to reduced expression of small conductance Ca2+-activated potassium channels (SK3), an association that appears to be attributable to a direct interaction between miR-499 and KCNN3 (Ling et al., 2017). The expression of the CACNB2-controlled L-type calcium channel β-subunit was reduced in the atria of patients with long-term persistent AF, while inhibition of miR-499a significantly enhanced the expression of CACNB2 in cultured atrial cells (Ling et al., 2017). In addition, by targeting CACNA1C, miR-499 could modify the expression of the L-type Ca2+ channel Cav1.2 and reduce the activity of ICa-L (Ling et al., 2017).

Although miR-499 manipulation emerged as a target for atrial electrical reverse-remodeling, this strategy is far from perfect, since miR-499 is also modified in other pathologies, including systemic lupus erythematosus or stroke (Wang M. et al., 2019; Jia et al., 2020; Banach et al., 2017).

#### 2.1.4 miR-26

Reduced atrial expression of miR-26 was highlighted in experimental models and in AF patients (Luo et al., 2013). Meanwhile, AF ablation seemed to restore miR-26 plasma levels (Dai et al., 2022). *In vitro* and *in vivo* studies demonstrated miR-26 involvement in regulation of KCNJ2 and, via increased activity of IK1, in action potential duration (APD) shortening (Luo et al., 2013). miR-26 knockdown increased AF propensity, while miR-26 overexpression reduced AF susceptibility, suggesting a potential therapeutic role for miR-26 manipulation (Luo et al., 2013).

Studies have shown that miR-26 is involved in normal tissue growth and development and up- or downregulation of miR-26 was associated with oncogenic or tumor-suppressive genes in various tumor types (Gao and Liu, 2011). Interventions for miR-26 upregulation to decrease AF susceptibility (Luo et al., 2013) could increase the risk of glioma (Gao and Liu, 2011), whereas miR-26 involvement in tissue growth and development may affect its accuracy as stable and specific biomarkers for AF.

#### 2.1.5 miR-31

In an experimental model, atrial-specific upregulation of miR-31 led to neuronal nitric oxide synthase (nNOS) depletion via dystrophin translation repression, contributing to APD changes and AF inducibility in mice (Reilly et al., 2016). In contrast, miR-31 inhibition restored APD and increased dystrophin expression and nNOS content in atrial myocytes from AF patients. In patients with AF, upregulation of miR-31 seemed to be limited to the atrial myocardium (Reilly et al., 2016). Thus, miR-31 modulation may be a safer approach for AF therapy.

#### 2.1.6 Other miRNAs

miR-21 overexpression has been associated with decreased ICa-L activity via decreased expression of CACNA1C and CACNB2 58 (Barana et al., 2014). Enhanced miR-21 transcription is likely to result from nuclear factor κB pathway activation, which reflects an increased inflammatory status that is commonly seen in AF (Barana et al., 2014). Recent research has linked low miR-29b levels with decreased Cx43 expression, suggesting that this miRNA may play a role in atrial proarrhythmic electrical remodeling (Lv et al., 2021). With advancing age, the expression of miR-133 appears to decrease, simultaneously with an increase in HCN2 and HCN4 expression (Li et al., 2015), which may contribute to an increase in AF susceptibility. Whether these changes are causally linked or represent independent responses to aging and AF remains unclear, highlighting the need for additional studies to elucidate these relationships. Increased miR-155 levels were also seen in the cardiomyocytes of patients with AF, and this was accompanied by a decrease in CACNA1C expression (Wang et al., 2021). Transfection of miR-155 in human induced pluripotent stem cells-derived atrial cardiomyocytes reduced ICa-L activity, while in mice with miR-155 overexpression APD decreased and there was an increase in AF vulnerability (Wang et al., 2021). Meanwhile, inhibition of miR-155 reversed the AF phenotype and the related electrical remodeling (Wang et al., 2021).

### 2.2 MicroRNA-mediated atrial structural remodeling

#### 2.2.1 miR-21

miR-21 is expressed in fibroblasts and is involved in cardiac hypertrophy by increasing ERK pathway activity via inhibition of protein sprouty homologue 1 (Spry1) (Thum et al., 2008). Considering the association between ERK activation and cardiac fibrosis (Thum et al., 2008), these results suggest that miR-21 could be involved in AF-related structural remodeling. Indeed, studies have shown a 2.5-fold higher left atrial expression of miR-21 in patients with AF compared to sinus rhythm controls (Adam et al., 2012). However, miR-21 is also expressed in non-atrial tissues and increased miR-21 expression has been observed in several other cardiac and non-cardiac diseases (Bautista-Sánchez et al., 2020; Yan et al., 2020; Bai and Bian, 2022; Liu et al., 2010).

The mechanisms by which miR-21 promotes AF are related to both electrical and structural atrial remodeling. Clinical studies demonstrated an association between extensive areas of low voltage in the left atrium of patients with AF and increased expression of miR-21 (Zhou et al., 2018). Experimental studies in mice with heart failure also associated atrial fibrosis and AF susceptibility with increased miR-21 expression (Cardin et al., 2012). Increased miR-21 levels were associated with increased amounts of atrial collagen and reduced expression of Spry1, connective tissue growth factor (CTGF), lysyl oxidase, and Rac1-GTPase, all of which have implications in atrial fibrosis (Adam et al., 2012). miR-21 could also play a role in atrial structural remodeling by activating the PI3K signaling pathway (Zhang et al., 2018). Meanwhile, miR-21 knockdown prevented left atrial structural remodeling, reduced fibrosis and AF persistence (Cardin et al., 2012), and doxycycline attenuated atrial remodeling by decreasing miR-21 and modulating miR-21-dependent pathways (Zhang et al., 2018). However, considering the involvement of miR-21 in many other pathologies, including cancers (Bautista-Sánchez et al., 2020), its use as a selective therapeutic target is difficult and requires careful study.

#### 2.2.2 miR-29

In patients with AF, atrial levels of miR-29b were found to be substantially lower compared to those of patients in sinus rhythm (Dawson et al., 2013). Unlike other miRNAs, miR-29 exerts antifibrotic effects (Van Rooij et al., 2008). miR-29 targets multiple extracellular matrix genes, including COL1A1, COL3A1, and fibrillin, and is involved in reducing cardiac (including atrial) fibrosis (Van Rooij et al., 2008). The decrease in miR-29b expression induced an increase in atrial expression of COL1A1 and in the amount of atrial collagen, confirming the antifibrotic effect of miR-29b (Van Rooij et al., 2008). In an atrial fibrosis model, administration of angiotensin II increased the amount of fibrotic tissue, reduced the expression of miR-29b, and increased atrial PDGF-B expression, while miR-29b overexpression reduced the degree of fibrosis, probably via miR-29b-mediated PDGF-B expression reduction (Lv et al., 2021). Another experimental study linked the antifibrotic effect of miR-29b to targeting transforming growth factor-β (TGF-β) receptor type-1 (TGFβRΙ) and inhibiting the Smad-2/3 pathway (Xinyuan et al., 2022).

However, miR-29 seems to also play a role in renal and pulmonary fibrosis (Cushing et al., 2011; Horita et al., 2021), which may affect its specificity as a biomarker for AF (Cushing et al., 2011; Horita et al., 2021; Malpeli et al., 2018; Xu et al., 2014). In addition, miR-29 downregulation has been reported in various tumors and autoimmune and hematological disorders (Horita et al., 2021; Xu et al., 2014).

#### 2.2.3 miR-126

In patients with AF, miR-126 levels were significantly lower than in patients in sinus rhythm (Wei et al., 2015). miR-126 overexpression has been shown to suppress Spred1 expression, increase ERK activity in primary bone marrow cells, and increase cytokine production (Ishizaki et al., 2011). However, it remains to be investigated whether these mechanisms are also active at the cardiac level.

#### 2.2.4 miR-133 and miR-590

In an AF model based on nicotine administration and rapid pacing, the two interventions induced atrial fibrosis via TGF-β1 and TGF-β receptor type-2 (TGFBR2), in parallel with a significant reduction in the expressions of miR-133 and miR-590, which target TGF-β1 and TGFBR2 (Shan et al., 2009). Although a direct causal link has not been established, these data suggest that low levels of miR-133 and miR-590 could contribute to atrial fibrosis. In an experimental study in rats with AF (Yao et al., 2020), suppression of MIAT, an inhibitor of miR133a-3p expression, reduced atrial fibrosis, inhibited the expression of genes that promote collagen deposition, and led to a decrease in CTGF and TGF-β1 levels. These changes were reversed by miR-133a-3p inhibition. However, implementation of such a strategy requires additional safety studies, given that miR-133 also intervenes in other diseases (Hua et al., 2021).

#### 2.2.5 miR-30

In an experimental model of AF in dogs, miR-30 levels were significantly lower 3 weeks after initiation of AF by rapid stimulation of the pulmonary veins (Li et al., 2012). Although atrial fibrosis was not evaluated in that study, previous studies have shown that miR-30 regulates CTGF, a key profibrotic protein, and controls structural changes in the extracellular matrix of the ventricular myocardium (Li et al., 2012). In a model of atrial fibrosis induced by angiotensin II, increased expression of the Snail-1 gene was associated with a decrease in miR-30a expression (Duisters et al., 2009). Meanwhile, an increase in miR-30a levels led to Snail-1 inhibition and decreased periostin expression, suggesting a direct involvement of miR-30a in atrial fibrosis (Yuan et al., 2015).

#### 2.2.6 miR-483

In patients undergoing coronary bypass surgery, preoperative levels of miR-483-5p were significantly higher in patients who developed postoperative AF compared to those who did not, suggesting that miR-483-5p levels may reflect the existence of an arrhythmogenic substrate (Harling et al., 2017). In studies with experimental pulmonary hypertension, miR-483 had important profibrotic effects (Zhang et al., 2020). The use of miR-483-5p as a biomarker for postoperative AF has also been proposed (Yuan et al., 2015). However, the presence of altered miR-483 expression in other pathologies and the ability of miR-483 to self-regulate its expression affects its specificity and sensitivity as an AF biomarker (Zhang et al., 2020; Matson et al., 2023).

#### 2.2.7 miRNA-155 and miRNA-24

Increased miR-155-5p and miR-24-3p levels were observed in pigs with AF, while ablation reduced the expression of these miRNAs in both pigs and patients with AF (Wang W. et al., 2019). These post-ablation changes were associated with nitric oxide (NO) reduction, suggesting the involvement of these miRNAs in endothelial NOS signaling pathway regulation (Wang M. et al., 2019). Unfortunately, neither miR-155 nor miR-24 are specific for AF, as their expression has been reported to be increased or decreased in many other pathologies (Wang W. et al., 2019).

#### 2.2.8 Other miRNAs

Studies have also associated increased miR-328 levels with atrial dilation and with increased left atrial volume index and voltage zone index (Soeki et al., 2016). Reduced miR-499-5p and increased SOX6 levels were also reported in rats with AF, while miR-499-5p overexpression attenuated atrial fibrosis in that model, probably via SOX6 reduction (Yao et al., 2020).

### 2.3 MicroRNAs-mediated atrial autonomic remodeling

The role of miRNAs in autonomic cardiac remodeling has been less studied. However, studies have associated various miRNAs with changes in the autonomic nervous system of the heart. miR-133a has been shown to regulate β1-adrenergic receptor transduction cascade, whereas miR-1/133a clusters regulate adrenergic control of cardiac repolarization (Ishizaki et al., 2011; Besser et al., 2014). It remains to be established whether these miRNAs also influence atrial repolarization.

miR-30 overexpression led to KCNJ3/Kir3.1 downregulation and reduced IK-Ach activity (Morishima et al., 2016). Since IK-Ach is involved in the regulation of AERP, miR-30 could affect the occurrence of AF (Morishima et al., 2016). In a canine model, atrial tachypacing increased AF inducibility and mean nerve density in the superior left fat pads. Overexpression of miR-206 additionally increased nerve density, while anti-miR-206 exerted opposite effects (Zhang et al., 2015). The mechanisms that explain this hyperinnervation are related to an increase of reactive oxygen species, coupled with a decrease in superoxide dismutase 1 induced by miR-206 (Zhang et al., 2015).

Recently, bioinformatics analysis of miRNA-predicted target genes suggested that miR-155-5p and miR-302a-3p could regulate nerve growth factor (NGF) signaling (Tran et al., 2019). The same study demonstrated that the two miRNAs are also associated with left atrial epicardial adipose tissue in AF patients. Given that the epicardial adipose tissue secretes cytokines and growth factors, including NGF, and that NGF can increase the activity of ganglionated plexi, located in the epicardial fat, NGF could represent a link between miR-155-5p and miR-302a-3p and proarrhythmic autonomic remodeling (Tran et al., 2019).

### 2.4 MicroRNAs as biomarkers in atrial fibrillation

The onset and progression of AF has been associated with numerous risk factors and with altered levels of several biomarkers. However, none of these risk factors and biomarkers have proven convincing reliability for AF diagnosis or prediction, especially since they are also involved in other cardiac and non-cardiac conditions (Kornej et al., 2020). Significant efforts have been made to develop risk scores to identify patients prone to AF, but their clinical role is not convincing (Olivia et al., 2019). The multifactorial etiology and the lack of risk factors for AF in certain patients are likely to contribute to the low reliability of such risk scores (Kornej et al., 2020; Olivia et al., 2019; Fan et al., 2019).

Accumulating studies have identified numerous miRNAs that are differentially expressed in the cardiac tissue of patients with AF compared to those in sinus rhythm, suggesting that they could be used as diagnostic markers for AF (Table 1). Although promising, this strategy involves sampling of the atrial tissue, which is not feasible in most AF patients. Subsequent research has thus focused on identifying miRNAs for which the circulating levels reflect their atrial expression. Due to their ability to associate with various microstructures such as exosomes, microvesicles, and apoptotic bodies, miRNAs exhibit remarkable stability in plasma (Takahashi et al., 2017). Also, they are often bound to proteins and high-density lipoproteins, which gives them protection against RNase enzymes (Takahashi et al., 2017). Studies demonstrated modified expression levels of several miRNAs, both in the left atrium and in the peripheral blood of patients with AF compared to those in sinus rhythm (Wei et al., 2015; Ishizaki et al., 2011). Plasma levels of miR-29b, miR-21, miR-328, and miR-150 seem to be modified in patients with AF compared to those without AF (Soeki et al., 2016; Dawson et al., 2013; Nishi et al., 2013). In patients with AF, miR-126 serum levels were found to be lower than in the control group (Wei et al., 2015). However, not all circulating miRNAs reflect their atrial expression. In a study performed in patients with postoperative AF (POAF), although in the atrium there was a difference between miR-208a expression in patients who developed POAF and those who did not, serum levels of miR-208a were not significantly different between the two groups (Harling et al., 2017). When analyzing the whole blood, among the 385 miRNAs evaluated in patients with AF, only miR-328 remained significantly associated with AF after adjusting for risk factors and RNA concentration and quality (McManus et al., 2014).

**TABLE 1:** Key microRNAs investigated as biomarkers in atrial fibrillation.

miRNA	Study subjects	Stage of disease	Sample type	Expression change	References
miR-1	Canines	Chronic AF	Left atrial appendage tissue	Upregulated	Chen et al. (2024)
miR-1-3p	Patients with cryptogenic stroke	Subclinical AF	Plasma	Upregulated	Benito et al. (2022)
miR-499	Canines	Rapid pacing induced chronic AF	Left atrial appendage tissue	Upregulated	Chen et al. (2024)
	AF patients	Paroxysmal AF	Plasma	Upregulated	Ma et al. (2017)
miR-21	AF patients	Persistent AF	Plasma	Upregulated	Chen et al. (2021)
miR-328	Spontaneously hypertensive rats	Paroxysmal AF	Left atrium and whole blood	Upregulated	Scridon et al. (2023)
	AF patients	Paroxysmal, persistent and permanent AF	Whole blood	Upregulated	McManus et al. (2014)
miR-483	AF patients	Post-operative AF	Serum	Upregulated	Harling et al. (2017)
miR-29b	Dogs	Congestive heart failure -related AF	Atrial tissue	Downregulated	Dawson et al. (2013)
	AF and chronic heart failure patients	Persistent AF	Plasma	Downregulated	Dawson et al. (2013)
	AF patients	Persistent AF	Atrial tissue	Downregulated	Dawson et al. (2013)
miR-30	Canines	Rapid pacing induced sustained AF	Atrial tissue	Downregulated	Li et al. (2012)
miR-133	Canines	Rapid pacing induced sustained AF	Atrial tissue	Downregulated	Li et al. (2012)
miR-208b	AF patients	Paroxysmal and persistent AF	Serum	Upregulated	Paul et al. (2021)
	Myocytes isolated from AF patients	Chronic AF	Atrial myocytes	Upregulated	Cañón et al. (2016)

AF, atrial fibrillation.

Studies, including from our team, demonstrated that atrial remodeling precedes the occurrence of AF (Șerban et al., 2019). Considering that several miRNAs have been associated with atrial remodeling, evaluating these miRNAs at an early stage could help identify patients predisposed to AF. In patients undergoing coronary bypass grafting, miR-483-5p atrial expression level was higher in patients who developed POAF than in those who did not. More importantly, the peripheral level of miR-483-5p was also significantly higher in POAF compared to control patients even before surgery (Harling et al., 2017). In a rat model of AF, we have also shown that changes in miR-328 expression preceded the occurrence of AF (Scridon et al., 2023). These results suggest that dynamic changes in peripheral miR-483-5p and/or miR-328 levels could emerge as biomarkers for AF prediction. Unfortunately, none of these miRNAs are specific for AF (Wang et al., 2023; Matson et al., 2023).

The potential role of miRNAs as biomarkers of AF recurrence after cardioversion or catheter ablation has also been investigated. miR-21 serum levels negatively correlated with ablation success at 1-year follow-up, suggesting that miR-21 levels could stratify patients according to the risk of recurrence and help plan more extensive ablative treatment in patients at high risk of recurrence (Zhou et al., 2018). miR-155 levels measured post-cardioversion also proved useful for AF recurrence risk evaluation (Zhang et al., 2019).

Also, miR-22-3p and miR-107 levels were significantly higher and miR-146a-5p levels were significantly lower in patients with AF who had an increased risk of major adverse cardiovascular events (Rivera-Caravaca et al., 2020). Adding these miRNAs to the 2MACE score increased its predictive ability in AF patients (Rivera-Caravaca et al., 2020). Although these hypotheses need further confirmation from larger-scale studies, they can be a starting point for establishing miRNAs as biomarkers for major cardiovascular events in patients with AF. Many other miRNAs have or are currently being studied in the context of FA, indicating a rapidly expanding field that holds significant promise for the discovery of new biomarkers.

### 2.5 MicroRNAs as therapeutic targets in atrial fibrillation

Studies have demonstrated that changes in miRNA expression can induce a substrate that promotes AF onset and/or maintenance, while restoring normal miRNA expression can reduce AF risk (Table 2).

**TABLE 2:** Effects of *in vivo* manipulation of microRNAs associated with atrial fibrillation.

miRNA	Species	miRNA manipulation	Effect	References
miR-1	Rabbit	Overexpression: lentivirus construct containing miR-1 precursor sequence	KCNE1, KCNB2 downregulation, AERP shortening, increased *I*_Ks_ activity, increased AF inducibility	Jia et al. (2013)
	Rabbit	Knockdown: lentivirus construct containing anti-miR-1 oligonucleotide	Alleviated KCNE1 and KCNB2 downregulation and AERP shortening, increased *I*_Ks_ activity	Jia et al. (2013)
miR-21	Rat	Knockdown: miR-21 antagomiR	Reduced atrial fibrosis and AF	Cardin et al. (2012)
miR-26	Mouse	Overexpression: adeno-associated virus containing miR-26a mimic	KCNJ2/Kir2.1 expression suppression, reduced AF	Luo et al. (2013)
	Mouse	Knockdown: adeno-associated virus containing antimiR-26a sequence	Increased KCNJ2/Kir2.1 expression, increased AF	Luo et al. (2013)
miR-29b	Mouse	Knockdown: adeno-associated virus containing antimiR-29b sponge	Increased atrial COL1A1 and collagen content	Dawson et al. (2013)
miR-328	Dog	Overexpression: adenovirus infection containing miR-328 precursor	Diminished L-type Ca^2+^ current activity, shortened atrial APD, enhanced AF	Lu et al. (2010)

AERP, atrial effective refractory period; AF, atrial fibrillation; APD, action potential duration.

Decreased expression of miRNAs associated with AF can be addressed using synthetic miRNA duplex approaches (miRNA mimics) or virus-mediated miRNA transfer (Ishida and Selaru, 2013). miRNA mimics have been used in experimental studies to evaluate the impact of miR-9, miR-23a, and miR-34a overexpression on the mechanisms involved in AF, but the vast majority have not been yet tested *in vivo* (Wiedmann et al., 2022). Importantly, *in vivo* studies that have used this strategy for other pathologies have not reported significant adverse reactions (Lhamyani et al., 2021). Lentivirus and adenovirus transfection containing precursors of miR-1 and miR-328 were used to increase the expression of these miRNAs, while an adeno-associated virus containing miR-26a mimic was used to increase the expression of miR-26a (Table 2) (Jia et al., 2013; Lu et al., 2010; Luo et al., 2013). Although the use of cardiotropic adeno-associated virus-mediated transfer seems safe in terms of extracardiac effects, unwanted immune or inflammatory responses and relatively low load capacity remain the main disadvantages of this method (Dawson et al., 2013; Ishida and Selaru, 2013).

For miRNAs with increased levels involved in AF, studies used three strategies to decrease miRNA levels: antimiRs, miRNA sponges, and miRNA erasers (Ishida and Selaru, 2013). Numerous studies have demonstrated the therapeutic potential of antimiRs in AF (Table 2), without major adverse reactions (Jia et al., 2013; Lu et al., 2010; Cardin et al., 2012). However, considering the systemic delivery, antimiRs could have extracardiac adverse effects (Ishida and Selaru, 2013). miRNA sponges and miRNA erasers seem to be safer from this point of view. In AF, decreasing the expression of miR-29b and miR-378 using a sponge-based strategy had no adverse reactions (Dawson et al., 2013; Wu et al., 2023). However, controlling the administered dose for successfully inhibiting the miRNA of interest is challenging (Ebert and Sharp, 2010). A much more specific approach involves the use of single-stranded masking oligonucleotides (miR-masks) (Ishida and Selaru, 2013). The advantage is that a specific mRNA target is blocked, while the other miRNA targets are not affected. miR-masks were used to study the effect of miR-26 on KCNJ2/KIR2.1 expression in AF, without adverse effects (Luo et al., 2013).

Experimental studies have shown a potential therapeutic role for miRNA-based approaches in AF. However, their long-term efficacy, safety, and pharmacokinetics in humans remain unknown. Future studies will have to elucidate if the results observed in experimental studies are also applicable in humans and if these approaches are sufficiently safe for clinical use.

## 3 Gaps in knowledge and future research

Identification of reliable AF diagnostic and predictive biomarkers is an area of ongoing research. The diagnosis of AF is established based on the identification of a typical arrhythmic episode on an ECG tracing. However, AF is often intermittent and asymptomatic. Hence, it often escapes detection, exposing patients to increased risk of complications. Readily available diagnostic AF biomarkers could identify patients in whom AF (ECG-based) diagnostic strategies should be intensified. This would be of particular interest in patients at risk of stroke, in whom AF diagnosis will be followed by prophylactic anticoagulation. In parallel, a reliable AF predictive biomarker could guide AF screening strategies and intensify substrate-modifying approaches to reduce AF. Such biomarkers could also guide antiarrhythmic therapies and improve selection for catheter ablation, reducing the rates of complications and costs. miRNAs are seen as highly promising in these regards, and several miRNAs have been associated with the presence and recurrence of AF. However, to date, with the exception of miR-31, no miRNA has been identified as being specific for AF, and the predictive ability of miRNAs identified so far is rather modest. More will have to be done to identify miRNAs specific for AF and validate them as AF biomarkers. Maybe the solution will come from a combination of multiple miRNAs with or without protein biomarkers on the same panel. Given the complexity of AF, such a tool would provide a more comprehensive picture of AF and could improve diagnostic accuracy.

Currently available antiarrhythmic drugs have limited efficacy and high side-effect profiles. Although promising in the preclinical studies, most upstream therapies have failed to show similar success in the clinical trials. Given their contribution to AF pathogenesis, miRNAs could represent the key to a novel therapeutic approach. Experimental studies are again very promising in this regard, although no clinical data is available at this point. Given the complexity and the multitude of mechanisms in which miRNAs are involved, miRNA manipulation could influence several pathophysiological pathways involved in AF, which places them as potential multifunctional therapeutic strategies in AF. However, the same features of miRNAs raise concerns regarding the potential off-target effects of miRNA manipulation, which could affect cardiac regions other than the atria or even organs other than the heart. For instance, miR-328 levels were significantly higher in animals with AF than in those without (Scridon et al., 2023), suggesting that miR-328 could represent a possible target for AF therapy. miR-328 is, however, a known tumor suppressor, and decreased miR-328 levels have been reported in several types of cancers, precluding its inhibition as a potential therapeutic approach for AF. Similar features are also seen for other miRNAs associated with AF. Strategies designed to specifically target atrial miRNA expression will probably provide the solution, but technical challenges remain to be overcome.

With the advent of miRNAs, we may have discovered the Holy Grail. However, extensive investigations and additional research are still needed before miRNAs become therapeutic targets or biomarkers in AF. Collaboration between multidisciplinary teams that include academic researchers, clinicians, biotechnologists and pharmaceutical companies is essential for the discovery of the specific miRNA or a panel of miRNAs with potential clinical utility. Performing more robust preclinical studies that use sensitive and specific miRNA detection methods are imperative. Many studies have used microarray techniques, which is a semi-quantitative method with high rates of false negative and positive results. The use of quantitative methods (e.g., qRT-PCR) could improve our knowledge about miRNA involvement in AF. The limitations of the clinical studies performed so far cannot be overlooked. The small number of patients and the high inter-individual variability in terms of sex, age, drug therapies, and concomitant conditions contribute to the inconsistencies of the results. Large multi-center datasets and registries will be needed to identify AF-specific miRNAs that could be used for AF prediction, diagnosis, and therapy, if such miRNAs exist. Last but not least, the great efforts of consortia are crucial for the standardization of experimental protocols. They ensure the experimental protocols reproducibility and the miRNAs validation in extended cohorts of patients, thus accelerating the use of miRNAs in clinical practice. The demonstrated success of other miRNAs that have proven effective in the treatment of other diseases such as cancer offer hope for similar achievements in AF, emphasizing the importance of comprehensive preclinical studies followed by rigorous clinical trials.

## 4 Conclusion

MicroRNAs play an essential role in regulating structural, electrical, and autonomic remodeling in AF. Experimental studies have shown that miRNA manipulation could prevent or even reverse pathological remodeling in AF. Clinical and preclinical studies suggest that changes in miRNA levels in blood or atrial tissue could emerge as novel biomarkers for the diagnosis and/or prediction of AF. However, it remains to be determined if and which of the miRNAs associated with AF have sufficient specificity and sensitivity to be used as AF biomarkers in clinical practice. Further research is also needed to establish if therapeutic strategies based on miRNA manipulation could emerge as effective and safe approaches for patients with AF.
